# Editorial: Inequalities in COVID-19 healthcare and research affecting women

**DOI:** 10.3389/fgwh.2023.1150186

**Published:** 2023-03-10

**Authors:** Vijay Kumar Chattu, Lakshmi Surya Prabha Manem, Behdin Nowrouzi-Kia, Kelly Jane Thompson, Hamid Allahverdipour, Sanni Yaya

**Affiliations:** ^1^ Department of Occupational Science & Occupational Therapy, Temerty Faculty of Medicine, University of Toronto, Toronto, ON, Canada; ^2^ Center for Interdisciplinary Research, Saveetha Dental College, Saveetha Institute of Medical and Technical Sciences, (SIMATS), Saveetha University, Chennai, India; ^3^ Department of Community Medicine, Faculty of Medicine, Datta Meghe Institute of Medical Sciences, Wardha, India; ^4^ Research & Development, Dr. NTR University of Health Sciences, Vijayawada, India; ^5^ George Institute for Global Health, University of New South Wales, Newtown, NSW, Australia; ^6^ Research & Development, Nepean and Blue Mountains Local Health District, Sydney, NSW, Australia; ^7^ Research Center of Psychiatry and Behavioral Sciences, Tabriz University of Medical Sciences, Tabriz, Iran; ^8^ Department of Health Education and Promotion, Tabriz University of Medical Sciences, Tabriz, Iran; ^9^ School of International Development and Global Studies, University of Ottawa, Ottawa, ON, Canada; ^10^ The George Institute for Global Health, Imperial College London, London, United Kingdom

**Keywords:** gender, equity, inequalities, women, COVID-19, mental health, research, sexual and reproductive health (SRH)

**Editorial on the Research Topic** Inequalities in COVID-19 healthcare and research affecting women

This Research Topic, *Inequalities in COVID-19 Healthcare and Research Affecting Women*, presents a rich collection of articles from all over, including Africa, Asia, Australia, North America, and Europe. The Topic is a compilation of various articles addressing diverse topics such as gender gaps, violence against women, gender bias in research, postpartum care, accessibility to services, and so on, with a basket of policy options and recommendations.

The current COVID-19 pandemic is no exception, as women are disproportionately affected by the global crisis. Therefore, building fair, sustainable, and healthy societies requires understanding and attention to the impacts of both sex and gender on health outcomes. Although the lockdowns and stay-at-home orders are critical in limiting and preventing COVID-19 spread, they devastate vulnerable groups such as women and girls for gender-based violence (GBV). Besides, preventive confinement practices exacerbate many causes of or contributors to violence against women and girls (1). Additionally, COVID-19 has revealed that women are underrepresented in ongoing COVID-19 research publications, in the governance of epidemic management, and as authors of COVID research (2). In the same context, it was also highlighted that women are generally underrepresented as research participants in COVID research, and pregnant women were frequently excluded from research (3).

Despite the World Health Organization's (WHO) Executive Board recognizing the need of including women in decision-making for pandemic planning and response, women's representation is inadequate in COVID-19 policy domains at both national and global platforms. COVID-19 policy domains (4). The COVID-19 pandemic has disrupted especially the sexual and reproductive health services globally, resulting in many unwanted pregnancies, stillbirths, and maternal and neonatal deaths with negative impacts on mental health outcomes for women. Chattu et al. have highlighted that digital health equity must be included in health policies, particularly in remote areas to address equity, access, and affordability (5). Therefore, as argued by Singh et al. achieving equity and equality remains a bigger challenge (6), highlighting the pursuit of the WHO's principle, “No one is safe until everyone is safe” (7). Given this context, this Research Topic contains a total of 11 articles has 4 original articles, 2 brief research reports, 3 reviews, one opinion piece and one perspective, which are summarized below.

A study by Ahinkorah et al. highlighted the alarming statistics of gender inequalities in Sub-Saharan Africa and discussed various strategies to mitigate them and improve services for women and girls. To overcome household, educational, work/employment, and housing inequities, the authors emphasized that these interconnected disparities require broader policy actions to improve the current burden faced by many women. The review concludes to avoid/discourage attending mass gatherings and ensure face-masking with non-medical cloth-like masks, which are low-cost preventive measures to prevent the virus's spread among women.

Another study from northeast India by Padhye et al. addressed the challenges of accessing maternal health care amid the COVID-19 pandemic. In this explorative study through purposive sampling, the data was collected through telephonic interviews among pregnant women, health care providers, and the members of the village health sanitation and nutrition committees. The study found that women were spending out-of-pocket for some essential services despite accessing public health facilities. They further concluded that the major challenges, such as lack of transport facilities and medicines, resulted in a high proportion of Caesarian section deliveries and stillbirths. This study emphasized health systems’ preparedness and strengthening of community health centers to ensure affordable and quality, uninterrupted maternal health care services.

Similarly, another Indian study by Josyula et al. highlighted their work through the global consortium “Accountability for Informal Urban Equity” (ARISE). This study explored participants’ lived experiences of health-seeking behaviour, healthcare recourse, and the well-being aspects among the women waste pickers who belong to a marginalized community in urban India. The study highlighted the intersectionality of various factors such as gender, socioeconomic factors, cultural contexts, and other potential occupational hazards these women may face and provided specific recommendations to ensure healthcare access, safety and security.

Khan and David have addressed the growing rates of intimate partner violence in Trinidad and Tobago, a twin island in the Caribbean region. The paper highlighted that the COVID-19 pandemic created a milieu which is conducive to domestic violence surge as there is already an existing high prevalence rate aggravated due to confinement and possibly other lifestyle factors such as increased consumption of alcoholic products and other drugs by the males at home. Besides, they also cited that women suffering from domestic violence or abuse may be less inclined to seek care at a hospital because of fear of COVID-19 infection. Eventually, a social distancing strategy, albeit essential to prevent virus spread, may exacerbate the violence and keep it hidden. The authors emphasized that health care providers must tactfully screen for domestic violence during virtual encounters/telemedicine platforms by using safe words and trying to dissuade perpetrators by creating a supportive environment for women.

Salter-Volz et al. from the United States, have reported on the sex and gender bias in research related to COVID-19 clinical case reports during the pandemic. Their bibliometric analysis concluded that the majority (61%) had male first authors, and the case reports with male last authors were more likely to describe male patients. However, the reports with female last authors were more likely to include both sexes, highlighting potential biases in disseminating clinical information *via* case reports. This study also explores the inextricable influences of both sex and gender biases within the domain of biomedicine.

Another interesting German study by Liu et al. on the molecular and physiological aspects of the SARS-CoV-2 infection among pregnant women highlighted the possibility of in-utero transmission of the virus based on the evidence from placental infection and expression of viral entry receptors at the maternal-fetal interface. The researchers added that SARS-CoV-2 could further damage the placenta, cause maternal systemic inflammation, and hinder access to healthcare services during the pandemic.

This Research Topic also included the role of sex and gender in vaccine research and, in this context, the review by Vassallo et al. has highlighted the failure to recognize important sex and gender implications on the efficacy, safety, and implementation are detrimental for the global vaccine rollout in controlling the COVID-19 pandemic. The review concluded that there were missed opportunities to apply a sex and/or gender-sensitive lens in developing COVID-19 vaccines. Further, they emphasized improving data reliability, fostering public trust in immunization programs, reducing vaccination hesitancy, and boosting coverage. They further recommended that public health data collected through routine disease surveillance be sex/gender-disaggregated and be made available to the general public.

An Australian study on mental health and well-being of postpartum women by Christie et al. has explored mothers’ mental health, well-being, and health behaviors up to 12 months postpartum under COVID-19 level III and level IV restrictions in Australia. The research suggested that most postpartum mothers have normal mental health symptoms, and most of them are happy, at least for a good amount of the time, despite being worn out. Further, the study highlighted the critical role of health values in maintaining physical activity during leisure and promoting mental health through participation in virtual group exercises, community programs, and socializing with friends.

Another Australian mixed methods study by Henry et al. investigated the local maternity service providers about the impact of COVID-19 on domestic and family violence (DFV), mental health screening, and broader health service provision. Half the respondents felt the pandemic negatively affected the delivery of services, timeliness and quality of services to pregnant women, DFV and screening and management of mental health issues. The study also concluded that women who were at high risk due to their physical health, mental health, DFV, or other social issues were considered unsuitable for telehealth services.

In the same context, Demeke and Shibeshi have also assessed and discussed the intimate partner violence against women of reproductive age and associated factors in Northern Ethiopia. The results showed an alarming rate of psychological (35%), physical (15%), and sexual violence (15%) among the respondents. The study concluded the high rates of IPV were due to multiple factors such as level of education, smoking habits, and alcoholism among male counterparts and highlighted the role of empowering women and simultaneously educating and sensitizing the male partners through tailormade programs.

Another qualitative research study from Germany by Batram-Zantvoort et al. explored the first wave of the COVID-19 pandemic on maternal self-conception and mental well-being. The study investigated how women referred to and made sense of the dominant gender norms in their arrangements of daily life during the pandemic and how these beliefs have impacted their maternal self-conception. The interviews were analyzed and were seen through the lens of “intensive mothering” ideology and “ideal workers” norms. They found that mothers’ notions of guilt and their decrease in health link to dominant discourses on motherhood intersect with “ideal worker” norms which further amplify the burden of gendered health inequalities.

In conclusion, the pandemic has also shown an increase in gender-based violence and domestic abuse. There is a lack of attention to sex and gender in COVID prevention and treatment research. The impact on women, particularly the immediate risks that are associated with their roles on the front line of healthcare, social care and secondary impacts, such as intimate partner violence risks during the extended periods of social isolation/distancing. The pandemic has disrupted SRH services globally and impacted women's mental health and well-being. Therefore, this Research Topic addressing the inequalities of COVID-19 healthcare and research affecting women is a valuable addition to the existing knowledge base with some exceptional original research studies (including qualitative, quantitative, and mixed-methods), review articles, and evidence-based policy recommendations from different geographical regions.
